# Listeriosis – a retrospective study of 5 years on risk factors and clinical outcomes at a tertiary care hospital in Islamabad, Pakistan

**DOI:** 10.1099/acmi.0.001069.v3

**Published:** 2026-02-04

**Authors:** Farwa Zaheer, Muhammad Usman, Sania Raza, Nawwal Naeem Chaudhary, Waleed Babar, Ayesha Rahat, Salman Riaz, Madeeha Fatima

**Affiliations:** 1 Shifa International Hospital, Islamabad, Pakistan; 2 Bahria University College of Medicine, BUHSCI, Islamabad, Pakistan; 3 Watim Medical and Dental College, Islamabad, Pakistan; 4 Shifa College of Medicine, Islamabad, Pakistan

**Keywords:** blood culture, cerebrospinal fluid (CSF), immunocompromised, *Listeria monocytogenes*, listeriosis

## Abstract

**Background.** *Listeria monocytogenes* is a common foodborne organism identified as a causative agent of multiple clinical conditions in unique circumstances such as pregnancy and immunocompromise. It is a Gram-positive rod and a facultative anaerobic organism. This paper presents a study over a timeline of 5 years in retrospect and explores the incidence of listeriosis amongst patients of different age groups, along with its associated risk factors and clinical outcomes.

**Materials and methods.** This study was conducted in retrospect from June 2019 to June 2024 at Shifa International Hospital, Islamabad. Ninety-seven cases of listeriosis were identified. These cases were culture-positive listeriosis where the pathogen was isolated from various samples such as blood and cerebrospinal fluid. Important risk factors associated with the clinical presentations were also documented, which included diabetes mellitus, chronic kidney disease and malignancy. The mean±sd was calculated for the continuous variable. Frequency and percentage were calculated for categorical variables. Chi-square tests were performed to assess associations with mortality and foetal outcomes.

**Results.** A total of 97 culture-confirmed listeriosis cases, comprising 44 (45.5%) males and 53 (54.6%) females, were obtained. Fifteen of the females were pregnant. Fever was the most common presenting symptom across all groups, with pregnant patients also reporting abdominal pain, vomiting and foetal complications, while non-pregnant patients showed a wider range, including neurological, respiratory and gastrointestinal complaints. Of the 97 patients, 86 had comorbidities – most commonly hypertension and diabetes – while 15 total adult deaths occurred. Eight pregnancies resulted in foetal losses. Descriptive trends in pregnant patients suggested worse foetal outcomes with higher C-reactive protein, total leukocyte count and maternal comorbidities. Ampicillin-based regimens were the most frequently used treatments, and all isolates were sensitive to the tested antibiotics.

**Conclusion.** This study highlights how listeriosis poses substantial morbidity and mortality risk, especially in pregnant cases. There is also a critical data gap, emphasizing the need for better diagnostic strategies, timely and targeted interventions, awareness of the clinical team and public health surveillance to reduce the burden of this often-overlooked infection in Pakistan.

## Data Summary

Data were entered in an Excel sheet and were analysed by using the Statistical Package for the Social Sciences version 23.00. The mean±sd was calculated for the continuous variable. Frequency and percentage were calculated for categorical variables. A chi-square test was performed to find the association of presenting features of the patients with adverse outcomes. A *P*-value of less than 0.05 was taken as significant. All underlying data are presented in the article.

## Introduction

*Listeria monocytogenes* is an environmental as well as a foodborne pathogen commonly found at sites such as soil, water and foods such as dairy products. It is a Gram-positive bacillus and is a facultative anaerobic organism. *Listeria* carries the unique quality of being able to survive and replicate at low temperatures, making it a difficult organism to detect and eradicate, particularly in foods that are kept refrigerated and are consumed without heating up [1,2]. Systemic infections caused by *L. monocytogenes* are called listeriosis and are usually the result of contaminated food consumption. It carries a significant health risk, more so because of the vulnerable condition of the patients, as it causes diseases in extremes of age, pregnancy and immune compromise, including cancers, chemotherapy and immunoglobulin deficiency [3,4].

Listeriosis can present in many different ways and clinical symptoms. It can cause a mild gastrointestinal illness in some individuals while causing severe invasive infections in others, including meningitis, sepsis and encephalitis [5]. It has the ability to cross the blood-brain barrier and thus can be a very frequent causative agent for central nervous system (CNS) infections, particularly in extremes of age. This contributes to a significantly high mortality associated with this organism [6]. Timely diagnosis of clinical symptoms and identification of the pathogen are critical in improving morbidity and mortality by administering early treatment, especially in at-risk individuals [7].

There have been multiple studies on identifying associated risk factors of listeriosis and comorbidities which may make the patient more vulnerable to acquiring *Listeria*, such as diabetes mellitus (DM), chronic kidney disease (CKD), malignancy and chronic liver disease (CLD) [8,9]. An extremely at-risk group includes pregnant women, as the state of pregnancy makes the women vulnerable to extreme clinical manifestations of *L. monocytogenes*, and as this organism is also capable of crossing the placental barrier, adverse pregnancy outcomes such as preterm birth, miscarriage and sepsis of the neonate are often inevitable [10,11].

Newer studies have also explored the molecular mechanisms and genetic framework of *L. monocytogenes* in order to get better insights into this organism’s ability to be able to survive at extreme temperatures, to cross various barriers and to cause severe systemic manifestations [5, 12]. Although effective and targeted antimicrobials such as ampicillin and gentamicin are available for the treatment of listeriosis, the challenge faced by public health experts due to its incidence in certain groups remains significantly high and tough [12,13].

Exploring the molecular mechanisms of pathogenesis of *Listeria*, specifically in pregnant cases, there are multiple virulent genes which provide the organism an ability to invade host tissues and cross barriers leading to critical repercussions [14]. The surface proteins which consist of *Internalin A (inlA)* and *Internalin B (inlB)* accelerate the adhesion, as well as invasion of the bacterial cell into the trophoblasts and epithelial cells of the placental membranes. After this, the pore-forming toxin, referred to as *Listerolysin O* (*hly*), enables the escape from the phagosome while the *actin-assembly protein* (*actA*) leads to an increased intracellular motility and cell-to-cell spread [15]. All of these mechanisms hold critical importance in causing placental colonization, foetal infections and adverse outcomes. An understanding of these mechanisms can assist in designing better preventive and therapeutic strategies in order to achieve favourable outcomes.

This study explored in detail the risk factors associated with patients with culture-positive listeriosis. We also followed up with the patients to get an insight into the clinical outcomes and the success of treatment. We aimed to bridge the gap in understanding the various contributing factors to human *listeria* infection and to be able to provide evidence-based information that may prove beneficial in management, as well as prevention of this disease.

## Methods

This comprehensive retrospective study was conducted at the Department of Microbiology, Shifa International Hospital, Islamabad, from June 2019 to June 2024, for 5 years.

All patients’ data were included in the study after they had submitted a written consent for their data to be used and analysed for scientific and research purposes. The study was started after taking approval from the Institutional Review Board of Shifa International Hospital, vide reference number 263-24.

The hospital’s electronic medical record system was used to extract patients’ comprehensive data from the last 5 years. All cases that were confirmed to be listeriosis based on clinical data, as well as on the organism’s isolation on culture and sensitivity, were included in this study. Cultures were performed on samples such as cerebrospinal fluid (CSF) and blood. Duplicate samples were excluded from the study. Patients presenting with symptoms of listeriosis but not having positive culture results were also excluded.

A total of 97 *Listeria*-positive cases were identified. We noted the age and gender of all the patients. Data regarding comorbid conditions such as DM, CKD, malignancy and CLD were also noted. Serum inflammatory markers such as total leucocyte count (TLC) and C-reactive protein (CRP) were also noted. CSF and blood culture specimens were received in the microbiology lab from patients suspected of having listeriosis. The specimens were Gram-stained and cultured according to standard lab practices. Identification of any significant bacterial growth was done after an incubation of 24 and 48 h on blood agar (Oxoid, UK) plates. The growths were Gram-stained, and catalase, bile aesculin, reverse CAMP and motility testing were performed. Isolates that were Gram-positive rods (GPRs), testing positive for catalase, aesculin hydrolysis and reverse CAMP, and had tumbling motility were further confirmed for identification using the GPR card of Vitek 2 automated identification system (bioMérieux, France). Susceptibility testing was also carried out by Vitek 2. Patient outcomes with regard to recovery, lost to follow-up and pregnancy-related outcomes were also recorded and noted.

Molecular methods such as the PCR or Sequencing techniques were not employed in our study due to local budgetary constraints and non-availability of infrastructure for a molecular lab during the study period. In routine microbiology, identification is relied upon conventional culture techniques, Gram staining and biochemical testing, carried out with validated methods.

## Results

**Fig. 1.**
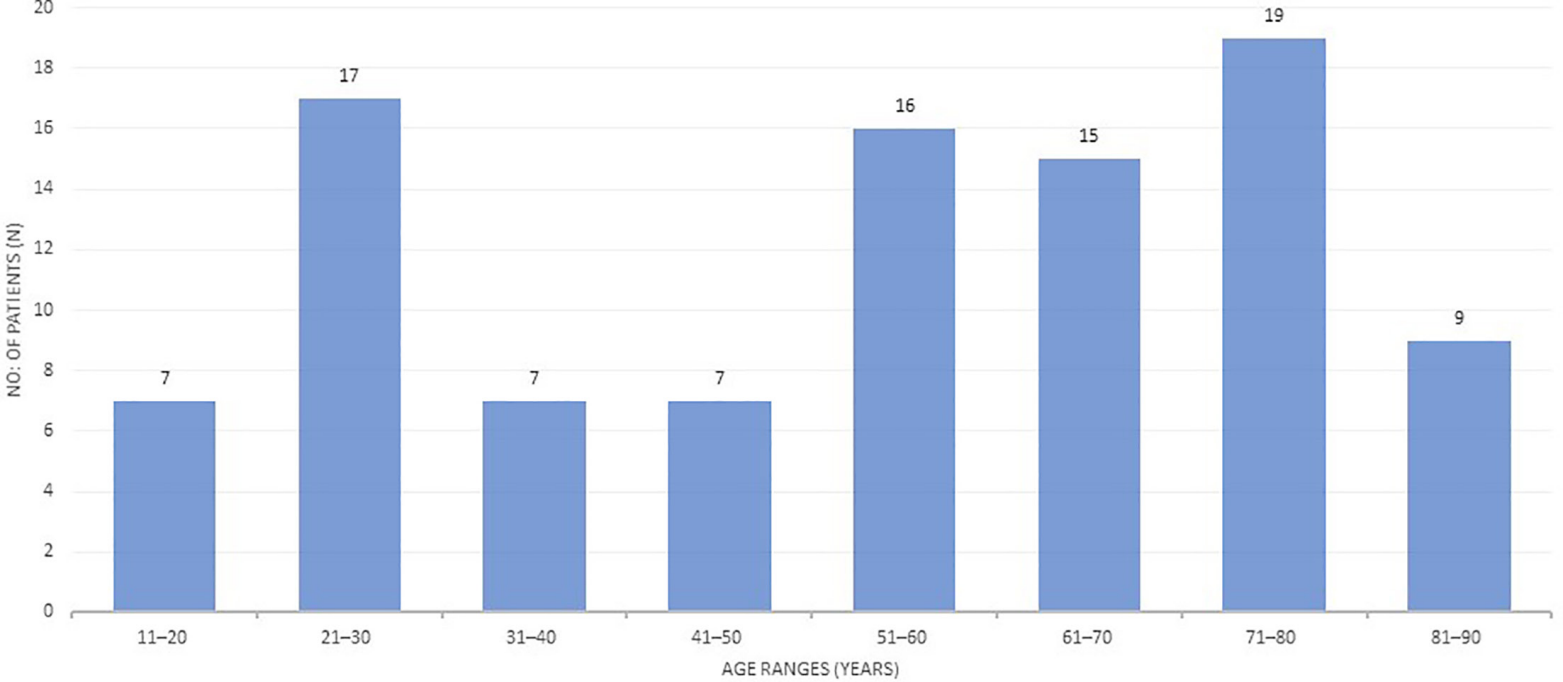
Age ranges of studied patients (*n*=97).

The data of 97 patients were included in this study after a thorough search for all cases reported as *Listeria* positive on culture and with significant clinical findings. It included 44 (45.5%) males and 53 (54.6%) females. Amongst female patients, 15 (28.3%) were pregnant at the time of culture-positive listeriosis, while 38 (71.6%) females were non-pregnant. We analysed the results by making two groups of patients, one of males and non-pregnant adult females and a second one for pregnant females. This was done for better prediction of foetal outcomes. The mean age of the patients was 58 years for non-pregnant adults and 29.7 years for pregnant females. The patients' demographics are described in Table 1, while groups of patients according to age ranges are shown in Fig. 1.

**Table 1.** Demographic characteristics of the patients (*n*=97)

Parameters	Number
Gender (n, %)	
Male	44, 45.5%
Female	53, 54.6%
Age (mean, years) Non-pregnant	58
Age (mean, years) Pregnant	29.7
Pregnant (n, %)	15, 28.3%

Presenting complaints were noted in non-pregnant, as well as pregnant, patients. Non-pregnant patients presented more frequently with fever, followed by neurological symptoms like headache, vomiting and altered consciousness or drowsiness. Other notable symptoms included seizures, respiratory symptoms (shortness of breath and cough), generalized weakness and neck stiffness. Less common complaints included gastrointestinal symptoms (diarrhoea and constipation), urinary symptoms and dermatological signs like rash or itching.

**Fig. 2.**
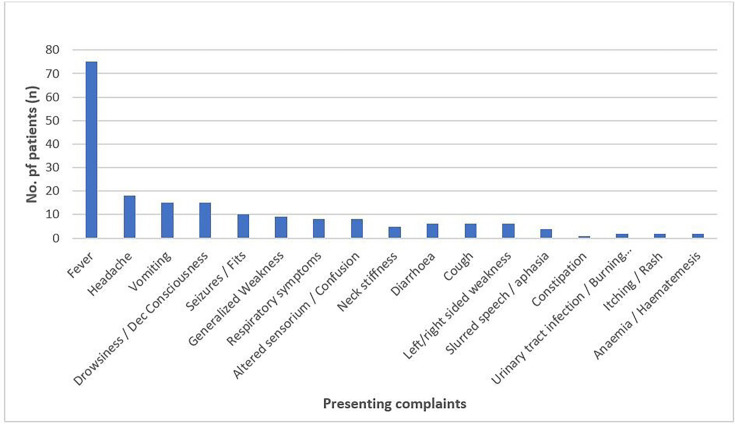
Presenting complaints of non-pregnant patients (*n*=82).

Fever was the most universal symptom, reported in all 15 pregnant cases. Other common complaints included abdominal pain in six patients and vomiting in four, indicating possible underlying infections or pregnancy-related complications. Labour-related issues such as early labour, preterm labour or labour pains were reported in four cases, while body aches, shivering or chills were noted in three, possibly reflecting systemic infection or febrile illnesses. Additionally, foetal complications, including reduced foetal movements and distress, appeared in three cases. Less commonly, neurological symptoms (e.g. imbalance, headache and neck stiffness) and respiratory complaints (e.g. shortness of breath and cough) were seen in two patients each. Figs2  3 highlight the various presenting complaints and the number of patients presenting with them.

**Fig. 3.**
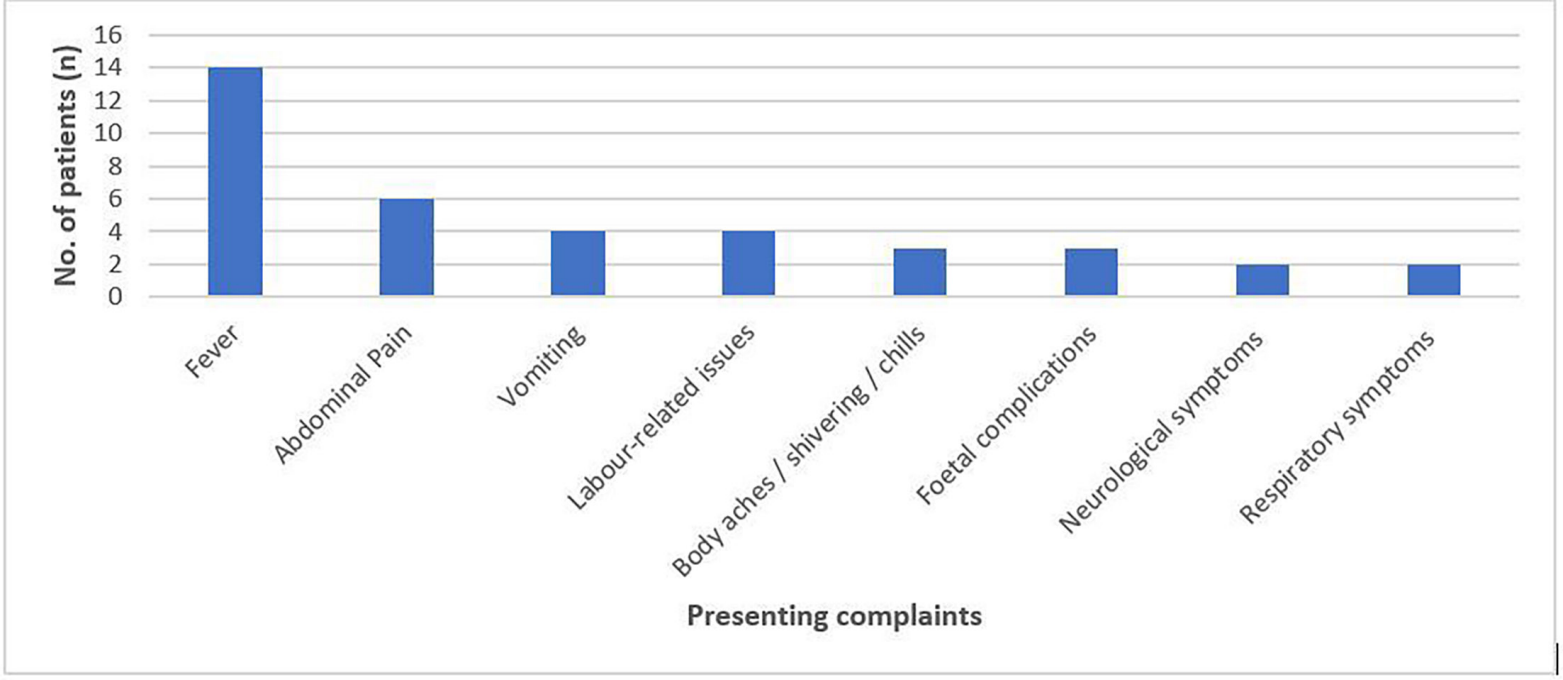
Presenting complaints of pregnant patients (*n*=15).

**Fig. 4.**
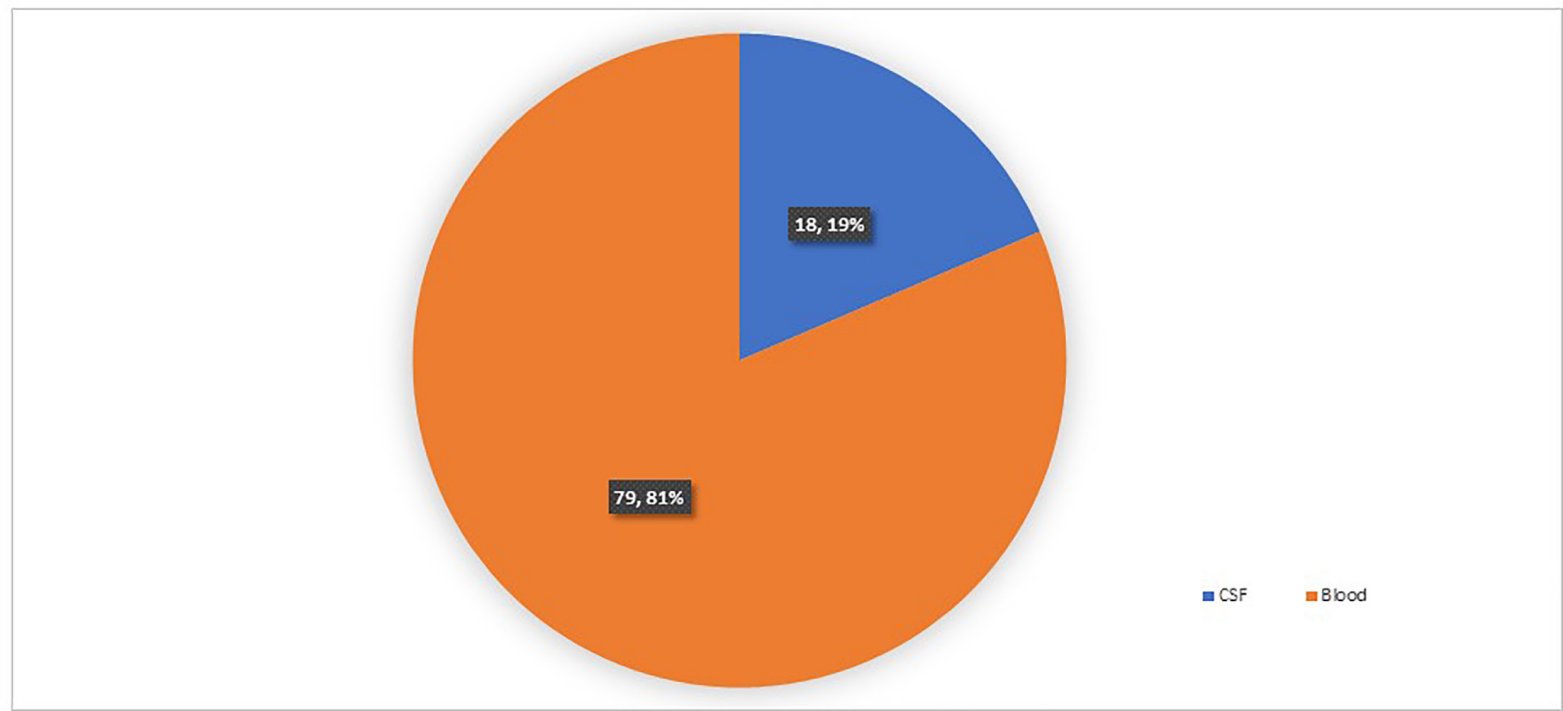
Samples received for isolation of *Listeria* (*n*=97).

These patients were diagnosed on positive culture for *Listeria* on either CSF or blood culture specimens. The number of each type of specimen received is given in Fig. 4.

Specimens were obtained from CSF through a lumbar puncture. Blood cultures were obtained either through peripheral venipuncture or through a central venous line, depending on the clinical setup.

**Fig. 5.**
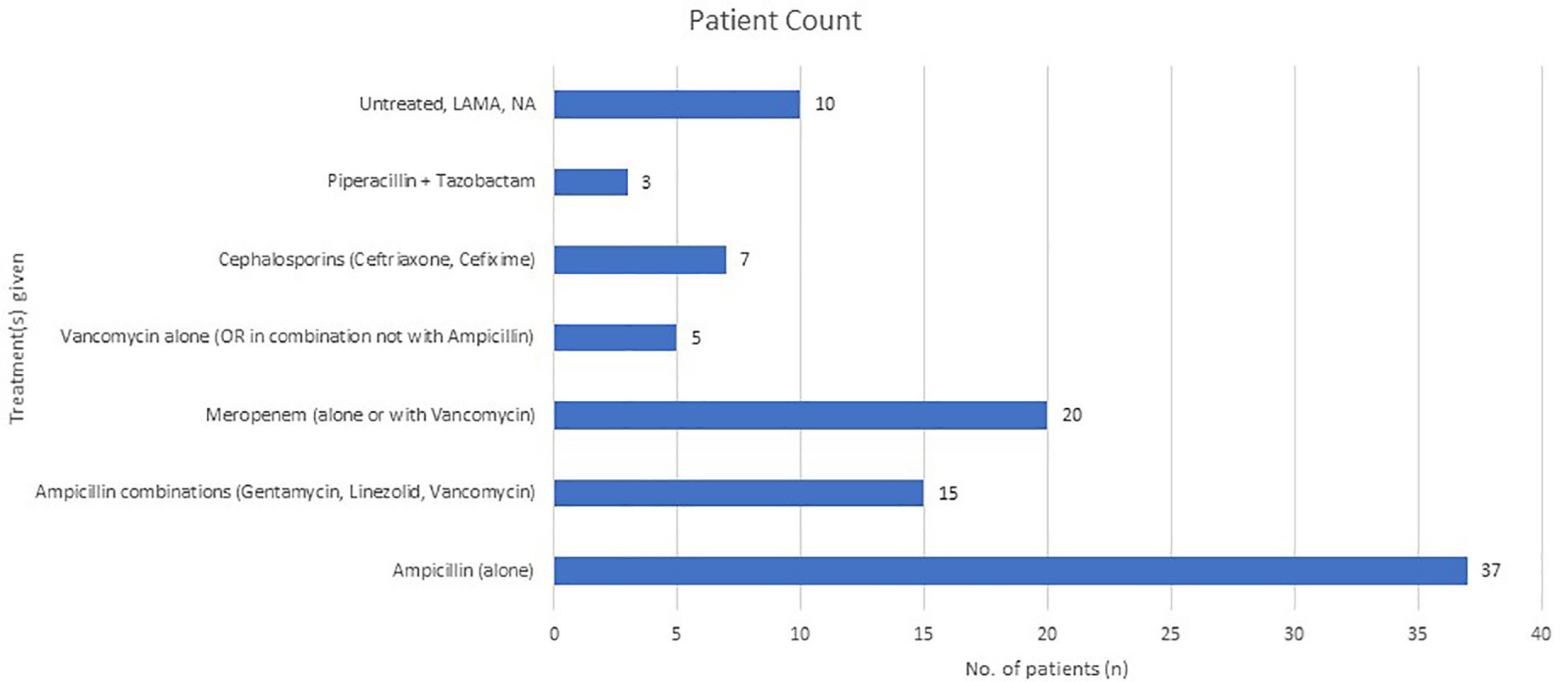
Treatment(s) given to patients with listeriosis (*n*=97).

The antibiotics tested for susceptibility resulted in the isolate being sensitive to the whole panel. The antibiotics tested included penicillin, ampicillin, cephalosporins (ceftriaxone and cefixime), vancomycin, piperacillin-tazobactam, gentamicin, linezolid and meropenem. Patients received a variety of treatment regimens, with ampicillin – either alone or in combination – being the most commonly administered antibiotic. Several patients were managed with dual therapies, including combinations such as ampicillin with gentamycin, vancomycin with ceftriaxone and ampicillin with linezolid. Broad-spectrum agents, such as meropenem and piperacillin-tazobactam, were also frequently used, particularly in more severe or resistant cases. A few patients were treated with vancomycin alone or in combination with meropenem, and in select cases, intrathecal vancomycin was administered. Cefixime and ceftriaxone were used in milder or outpatient scenarios. Notably, a small group of patients left the hospital against medical advice before completing therapy. Fig. 5 gives an overview of the treatments patients received in view of the all-sensitive antimicrobial susceptibility results.

**Fig. 6.**
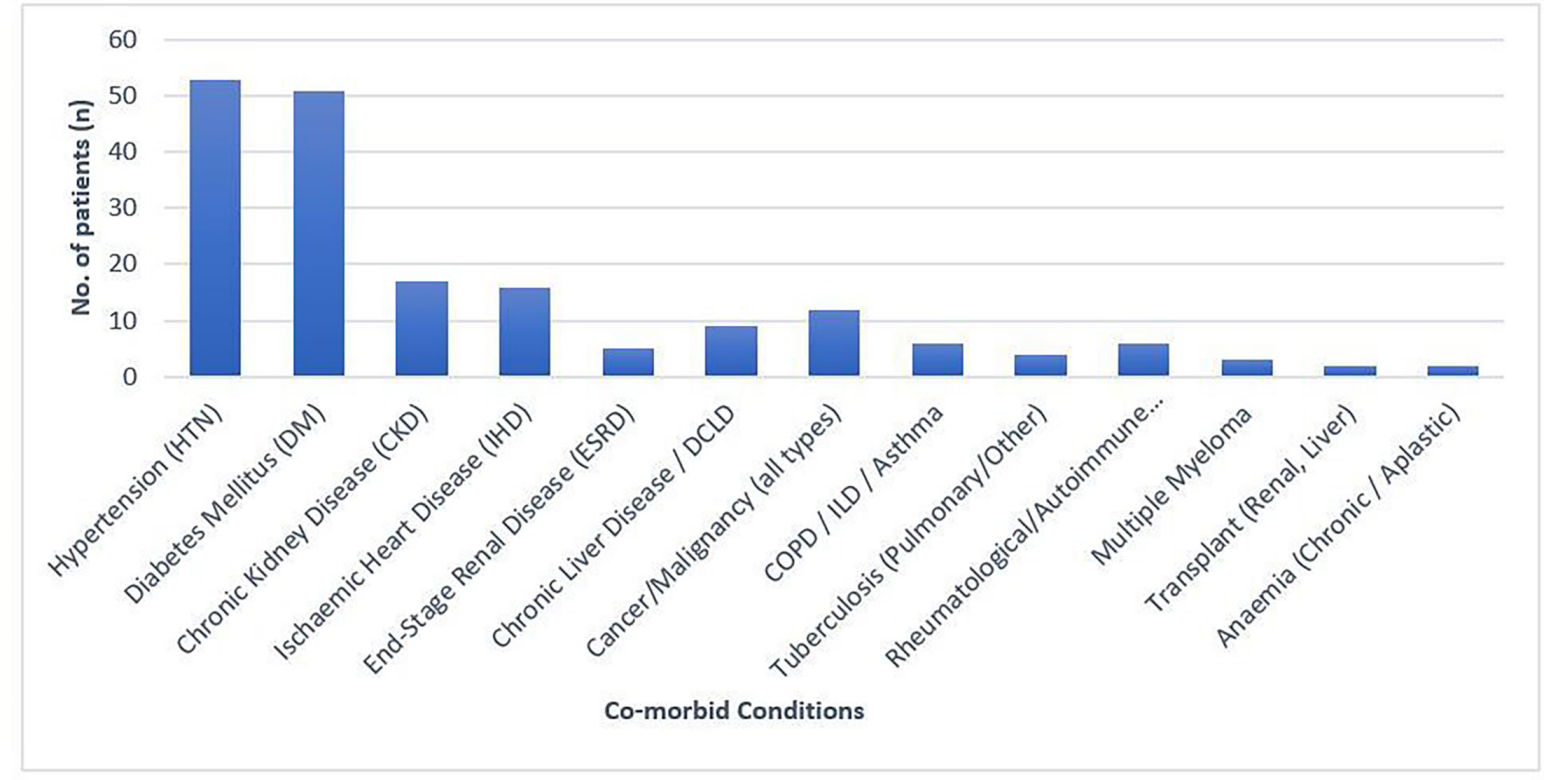
Different comorbid conditions and their occurrence (*n*=86).

Out of a total of 97 patients, 86 were found to have one or more comorbid conditions, while 11 had no underlying illnesses at the time of their diagnosis. These comorbidities spanned a wide clinical spectrum – from conditions like hypertension and diabetes to chronic illnesses such as malignancies, CKD and end-stage organ failure. A significant number of patients had hypertension and DM, making these the most prevalent risk factors. Other notable comorbidities included CKD, ischaemic heart disease and CLD. Additionally, some patients had a history of malignancy and end-stage renal disease. Respiratory disorders such as chronic obstructive pulmonary disease, interstitial lung disease (ILD) or asthma were observed in a few patients, while autoimmune or rheumatological conditions like rheumatoid arthritis or systemic lupus erythematosus (SLE) were also reported in several cases. Less frequent but clinically significant conditions included tuberculosis, multiple myeloma and organ transplant recipients. A small group of patients had chronic anaemia or other specific conditions like pregnancy-induced hypertension (PIH); gestational DM (GDM); haemolysis, elevated liver enzymes and low platelet count (HELLP) syndrome; or benign prostatic hyperplasia (BPH). The count of these comorbidities is specified in Fig. 6.

Along with comorbid conditions, we also noted serum TLC in microlitres (µl) and CRP values in milligrams per decilitre (mg dl^−1^). In the non-pregnant group of patients, a total of 29 (35.4%) patients had a TLC of less than 10,000 µl, while 47 (57.3%) patients had a TLC of more than 10,000 µl. The CRP values noted in this group were less than 100 mg dl^−1^ in 38 (46.3%) of patients, while more than 100 mg dl^−1^ in 32 (39.0%) patients. We were unable to trace the TLC and CRP values of approximately nine patients in this group.

In our set of 15 pregnant females, 10 (66.7%) had a TLC of more than 10,000 µl, while 8 (53.3%) had a CRP of more than 100 mg dl^−1^, highlighting the status of inflammatory response their body was in. The details are highlighted in Table 2.

**Table 2.** TLC and CRP values recorded in patients with positive *Listeria* culture (*n*=97)

Group of patients	TLC (µl)	No. of patients, n (%)	CRP (mg dl^−1^)	No. of patients, n (%)
Non-pregnant adults (*n*=82)	<10,000	29 (35.4%)	<100	38 (46.3%)
	>10,000	47 (57.3%)	>100	32 (39.0%)
	Not traced	6 (7.3%)	Not traced	11 (13.4%)
Pregnant females (*n*=15)	<10,000	5 (33.3%)	<100	7 (46.7%)
	>10,000	10 (66.7%)	>100	8 (53.3%)

In our analysis of 97 patients diagnosed with listeriosis**,** outcomes were categorized separately for pregnant and non-pregnant patients to highlight the distinct clinical trajectories within these groups.

**Fig. 7.**
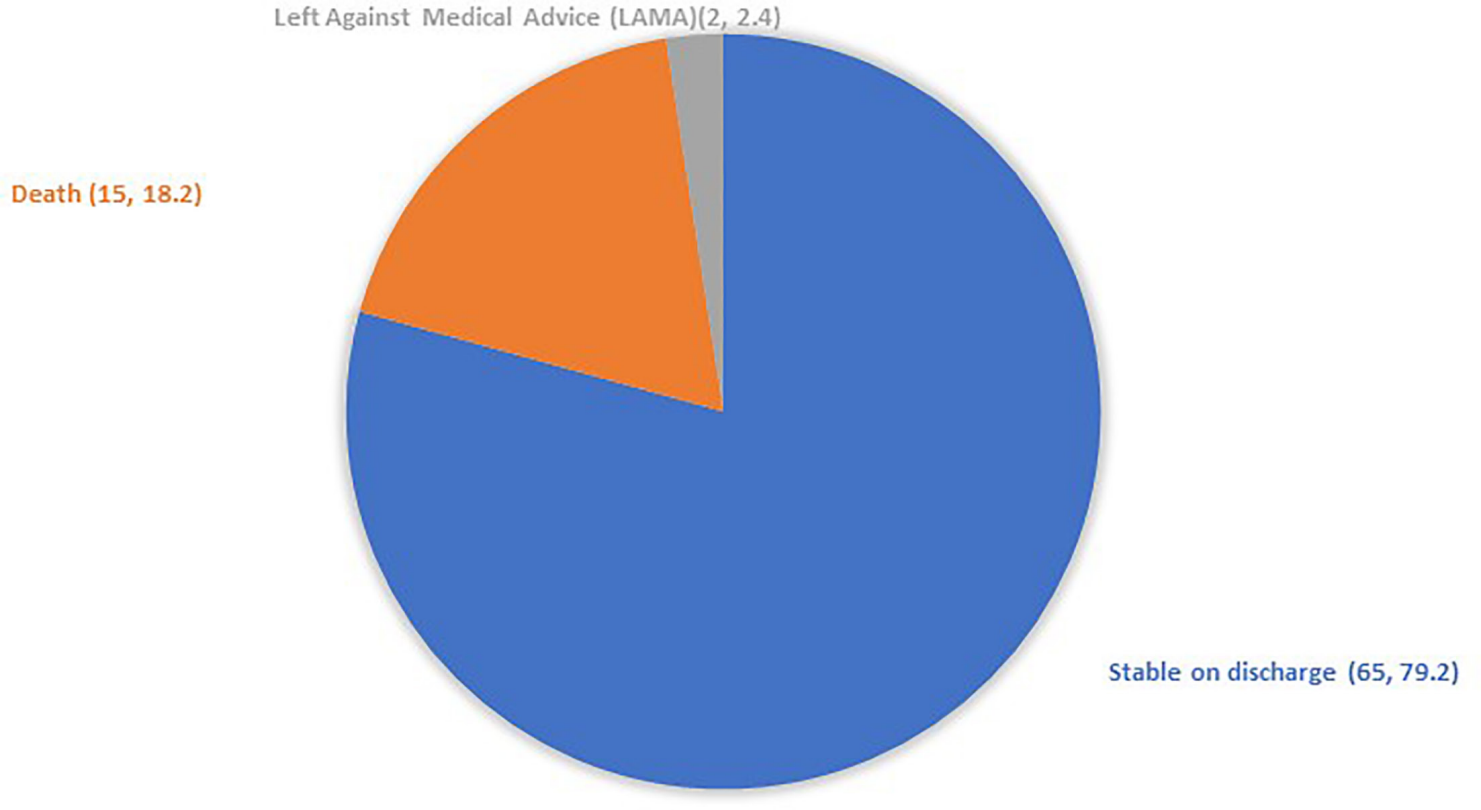
Non-pregnant patients' outcomes affected by listeriosis (n, %) (*n*=82).

For the non-pregnant patients, outcomes were grouped into successful treatment, death due to infection and leaving against medical advice, which are summarized in Fig. 7. Most non-pregnant patients responded well to therapy, while others were lost to follow-up, making final outcome assessment difficult in those cases.

A total of 15 deaths were recorded in this study. All of the patients who could not survive were carrying some comorbid conditions. The details of those patients are outlined in Table 3.

**Table 3.** Details of adult (non-pregnant) fatal cases of the study (*n*=15)

Patient	Age	Gender	Specimen	TLC	CRP	Comorbidity	Treatment
1	70+	F	Blood	>10,000	>100	ESRD, DM, HTN	Meropenem+vancomycin
2	10+	F	Blood	na	na	Liver failure	Vancomycin
3	50+	M	Blood	<10,000	<100	DM, HTN, hepatitis	Ampicillin
4	10+	F	Blood	na	na	None	Vancomycin+ceftriaxone
5	80+	M	Blood	>10,000	na	IHD, CKD, BPH	Meropenem
6	60+	M	CSF	na	na	IHD	Untreated due to death
7	70+	M	CSF	na	na	DM, HTN	Meropenem
8	60+	F	Blood	<10,000	>100	ILD, DM	na – death
9	50+	F	CSF	na	<100	HTN	na – death
10	70+	F	Blood	<10,000	>100	CKD, DM, HTN, IHD	na – death
11	20+	M	Blood	>10,000	>100	Multiple myeloma	na – death
12	70+	F	Blood	na	na	HTN, DCLD	na – death
13	10+	F	CSF	<10,000	na	None	na – death
14	60+	M	Blood	>10,000	<100	IHD	na
15	60+	M	Blood	<10,000	>100	HTN, HL	Untreated due to death

**Fig. 8.**
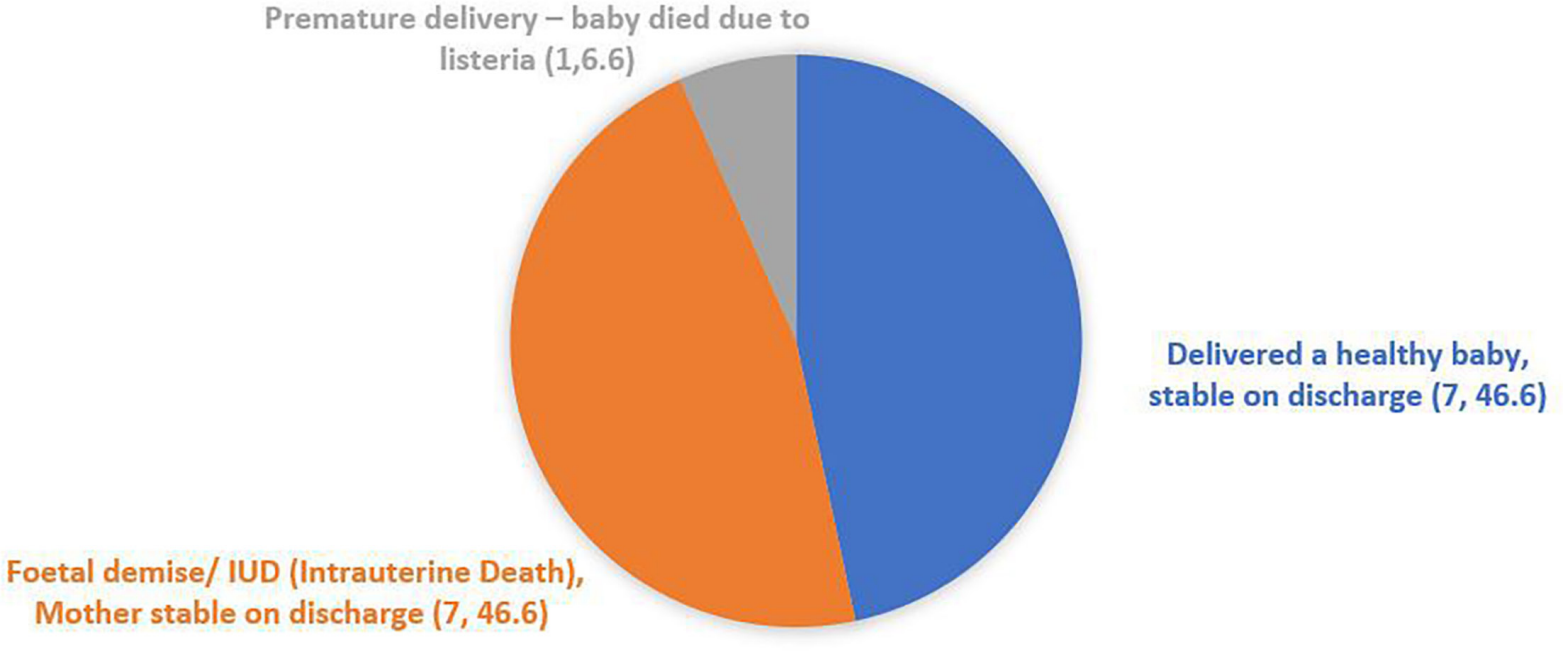
Pregnant patients' outcomes affected by listeriosis (n, %) (*n*=15).

Among the 15 pregnant patients, maternal survival was observed in all cases. In terms of foetal outcomes, seven pregnancies ended in healthy deliveries, while eight cases resulted in foetal demise, premature delivery with death, intrauterine death (IUD) or neonatal death. A summary of materno-foetal outcomes of the pregnant patients is presented in Fig. 8. Complete details of all pregnant *Listeria*-positive cases are outlined in Table 4.

**Table 4.** Details of all *Listeria*-positive cases in pregnant females (*n*=15)

Patient	Age	Specimen	TLC	CRP	Comorbidity	Treatment	Outcome
16	20–25	Blood	<10,000	>100	Fever	Ampicillin	IUD (second trimester)
17	30–35	Blood	<10,000	<100	Fever, abdominal pain	Ceftriaxone	Healthy
18	30–35	Blood	<10,000	>100	Fever, abdominal pain	Meropenem	Foetal death (third trimester)
19	35–40	Blood	>10,000	>100	Chronic anaemia	Meropenem	IUD (second trimester)
20	30–35	Blood	<10,000	>100	Nil	Piperacillin+tazobactam	Healthy
21	40–45	Blood	>10,000	>100	Fever	Ampicillin	Premature delivery – death
22	30–35	Blood	>10,000	<100	Nil	Meropenem	Foetal death (third trimester)
23	25–30	Blood	>10,000	<100	DM, PIH	Piperacillin+tazobactam	Foetal death (third trimester)
24	30–35	Blood	>10,000	<100	DM	Ampicillin	Healthy
25	20–25	Blood	<10,000	<100	SLE, CKD, HTN	Cefixime	Healthy
26	25–30	Blood	>10,000	>100	Nil	na	Healthy
27	30–35	CSF	>10,000	<100	Cerebellar abscess	Ampicillin	IUD (second trimester)
28	25–30	Blood	>10,000	>100	DM, IHD, HTN	Meropenem	Healthy
29	25–30	Blood	>10,000	<100	GDM	Piperacillin+tazobactam	Healthy
30	15–20	Blood	>10,000	>100	HTN, HELLP	Ampicillin+gentamycin	IUD (second trimester)

For all IUDs, no *Listeria*-related testing could be performed on the foetus due to social limitations. For the one premature delivery which eventually resulted in foetal demise at birth, CSF taken from anterior fontanelle yielded *Listeria* on culture. The healthy newborns were tested for *Listeria* through CSF culture taken from anterior fontanelle. All cultures tested negative for growth of *Listeria*.

For all *Listeria*-positive cases, we performed a chi-square test to find out any significant association of patient variables with adverse outcomes. The test was performed separately for non-pregnant and pregnant patients. Table 5 shows the chi-square test results for non-pregnant cases.

**Table 5.** Association between clinical parameters and mortality using the chi-square test – non-pregnant cases (*n*=82)

Variable	Chi-square (χ²) value	df	*P*-value
TLC category vs outcome	31.09	4	0.000
CRP category vs outcome	18.42	6	0.005
Comorbidities vs outcome	3.71	2	0.156
Treatment given vs outcome	33.05	2	0.000
Gender vs outcome	2.38	2	0.304
Age vs outcome	73.70	84	0.782

Mortality was significantly higher among patients with elevated TLC, raised CRP and those who did not receive treatment, while age, gender and comorbidities were not significantly associated with outcome. Among 78 patients with comorbidities, 11 (14.1%) died, compared with 2 of 4 (50%) without comorbidities. Mortality in patients with TLC <10,000 was 5 out of 29 (17.2%), versus 3 out of 47 (6.4%) in those with TLC >10,000; notably, patients with missing TLC data had a mortality of 83% (5 out of 6). Similarly, among patients with CRP >100 mg l^−1^, 4 deaths occurred (12.5%), compared with 3 out of 38 (7.9%) in those with CRP <100 mg l^−1^, and 5 out of 11 (45.5%) in those with unavailable CRP values. Mean age among deceased patients was 52.4±10.2 years, compared with 47.8±11.7 years in survivors. Deaths occurred in 8 out of 41 (19.5%) males and 5 out of 41 (12.2%) females. Overall, the non-pregnant adult cohort showed a trend towards increased mortality in patients with comorbidities, elevated inflammatory markers and older age. Patients with missing or unrecorded inflammatory markers often had poorer outcomes, likely reflecting more severe or unmonitored disease. Chi-square analysis confirmed that raised TLC (*P*<0.001), elevated CRP (*P*=0.005) and lack of treatment (*P*<0.001) were significantly associated with mortality, whereas no significant association was observed with age, gender or comorbidities.

In the pregnant cohort, women were younger (mean ~30 years). Despite most receiving treatment, foetal mortality was 53%, notably higher than adult mortality. Table 6 summarizes the association of maternal variables with foetal outcomes.

**Table 6.** Association between clinical variables of pregnant patients and foetal outcome (*n*=15)

Variable	Chi-square value	*P*-value
Age (continuous)	15.000	0.241
TLC category (<10,000 vs >10,000)	0.536	0.464
CRP category (<100 vs >100)	0.579	0.447
Comorbidity (yes/no)	0.077	0.782
Treatment given (yes/no)	1.224	0.268
Treatment regimen (ampicillin, meropenem, etc.)	6.295	0.391

No variable reached statistical significance, likely due to the small sample size (*n*=15). However, descriptive trends suggest that elevated inflammatory markers were more frequent among foetal deaths. Specifically, among mothers with CRP >100 mg l^−1^, foetal death occurred in 5 of 8 cases (62.5%), compared with 3 of 7 cases (42.9%) with CRP <100 mg l^−1^. Similarly, elevated TLC (TLC >10,000 cells per µl) was observed in 6 of 8 (75%) mothers who experienced foetal demise, compared with 4 of 7 (57%) among those with live births. Foetal death also occurred in 5 of 8 (62.5%) mothers with underlying comorbidities, compared with 3 of 7 (42.9%) without comorbidities. Almost all mothers (14 out of 15; 93%) received treatment, yet the only untreated mother had a foetal death. Mean maternal age was slightly higher in foetal death cases (30.6±5.2 years) compared with those with live births (28.8±4.7 years).

## Discussion

Although listeriosis is not a very frequently encountered infection, it remains an important challenge in healthcare settings due to its severe outcomes, especially when it is diagnosed in vulnerable patient groups such as neonates and the elderly, pregnant females, immunocompromised individuals and patients debilitated due to chronic diseases. *L. monocytogenes*, which is the causative agent, is able to survive in different environmental situations and carries the ability to cause invasive infectious conditions such as sepsis and meningitis [16,17]. Although there have been advancements in diagnostic strategies which led to timely interventions as well, the adverse outcomes associated with listeriosis remain high. Surveillance data from multiple centres report a resurgence in cases of *Listeria*, which can be attributed to longer life expectancy leading to more positive cases encountered in aged populations, high use of immunosuppressive therapies and better isolation or detection methods [18,19]. This detailed study significantly contributes to the existing literature by going through clinical presentations, demographic characteristics, comorbidities, treatment regimes and outcomes among 97 confirmed cases of listeriosis.

This study highlighted females being affected more, i.e. 54.6% of the total patients as compared to males, which is comparable to global data where females, especially pregnant ones, form a major risk group [20]. The mean age observed in these patients bears similarity to other reports, which suggests the typical bimodal distribution of listeriosis, affecting neonates and senior adults [10]. We identified 15 pregnant females, the majority of whom came with complaints of fever, mild to moderate abdominal pain and some with labour-associated complications. A poor foetal outcome was seen in almost 53% the cases, presenting with either IUD or foetal death despite survival of the mother. Recent data gave a very similar picture of adverse foetal outcomes which emphasized the typical tropism of *L. monocytogenes* for placental tissue and foetal membranes, eventually leading to non-favourable feto-maternal outcomes [21]. These outcomes also emphasize the need for timely recognition and management in obstetric cases. In France, a multicentre study was conducted, which reported a very high foetal loss rate of 29% in listeriosis, quite similar to our findings [22].

Among non-pregnant patients, including males, the most common presenting complaint was fever accompanied by either neurological symptoms or gastrointestinal manifestations. These observations mirror other studies conducted on non-pregnant adults, particularly those with chronic comorbidities, regarding their presentation with severe manifestations of listeriosis such as meningitis and septicaemia [23]. Almost 86 patients out of the 97 included in this study had one or more comorbid conditions. The most frequent ones were diabetes and hypertension, similar to findings from a 2021 meta-analysis highlighting how these conditions may serve as strong predisposing factors [24]. A case fatality rate of 16% has been observed in our patients, which is comparable to recent global studies estimating a mortality rate of 15–20% in invasive *Listeria* infections [17,25]. It is worth noting that all isolates were tested susceptible to first-line antimicrobials such as ampicillin and meropenem, reinforcing the continued effectiveness of these agents when used appropriately [7,26].

This study underscores the complex clinical spectrum of listeriosis and highlights the urgent need for increased clinical suspicion, particularly in high-risk populations. The disproportionately poor foetal outcomes despite maternal recovery emphasize the significance of early detection during pregnancy. The ongoing susceptibility of isolates to standard antibiotics is encouraging, but the relatively high mortality, especially among patients with comorbidities, requires strong clinical vigilance. Future directions should concentrate on enhancing hospital infection control measures, updating food safety regulations and improving early diagnostic capabilities, particularly in resource-limited settings where listeriosis remains underdiagnosed [3,27, 28]. Our findings support the necessity for continued epidemiological surveillance and patient-centred risk mitigation strategies.

## Limitations

There are a few limitations to our study that need to be acknowledged. The retrospective nature of this study does limit the ability to establish causality. Despite being a detailed study, conducted at a single centre, the results and findings may not be sufficient to be generalized to broader populations. We do feel that multicentre studies are needed to validate the findings of our study, as well as to highlight if there are any regional variations in epidemiology. Molecular mechanisms of *L. monocytogenes* pathogenesis, including its virulence factors and host cell interactions, can also be explored to offer valuable insights for the development of better treatment strategies.

## Conclusion

This detailed study presents an analysis of culture-positive and clinically proven cases of invasive listeriosis in Pakistan. Although very few similar studies can be found like this one in our region, they still highlight the significant impact *L. monocytogenes* can have on at-risk populations, particularly pregnant females, older adults, neonates and immunocompromised individuals. A few chronic comorbid conditions, such as DM and hypertension, were found as a common occurrence in our patients. Over 5 years, 97 cases tested positive for *L. monocytogenes* and revealed its potential to cause severe systemic and CNS infections, highlighting the importance of timely recognition and treatment. While all isolates tested showed a 100% susceptibility to standard antimicrobials, the high rate of adverse foetal outcomes stresses the urgent need for regular maternal screening and targeted preventive measures.

These findings do highlight a critical data gap, emphasizing the need for better diagnostic strategies, awareness of the clinical team, public health surveillance and food safety measures to reduce the burden of this often-overlooked infection in Pakistan.

## Abbreviations

BPH: benign prostatic hyperplasia
CKD: chronic kidney disease
CLD: chronic liver disease
CNS: central nervous system
CRP: C-reactive protein
CSF: cerebrospinal fluid
DM: diabetes mellitus
GDM: gestational DM
GPRs: Gram-positive rods
HELLP: haemolysis, elevated liver enzymes and low platelet count
ILD: interstitial lung disease
IUD: intrauterine death
PIH: pregnancy-induced hypertension
SLE: systemic lupus erythematosus
TLC: total leucocyte count
